# P-936. Incidence and Risk Factors for Ventricular Assist Device Infections: A Retrospective Review from the Middle East Gulf Region

**DOI:** 10.1093/ofid/ofae631.1127

**Published:** 2025-01-29

**Authors:** Mohamed Hisham, Noor Salmeh, Hajra Shah, Rania M El Lababidi, Bassam Atallah, Emna Abidi, Claude Afif

**Affiliations:** Cleveland Clinic Abu Dhabi, Abu Dhabi, Abu Dhabi, United Arab Emirates; Cleveland Clinic Abu Dhabi, Abu Dhabi, Abu Dhabi, United Arab Emirates; Cleveland Clinic Abu Dhabi, Abu Dhabi, Abu Dhabi, United Arab Emirates; Cleveland Clinic Abu Dhabi, Abu Dhabi, Abu Dhabi, United Arab Emirates; Cleveland Clinic Abu Dhabi, Abu Dhabi, Abu Dhabi, United Arab Emirates; Cleveland Clinic Abu Dhabi, Abu Dhabi, Abu Dhabi, United Arab Emirates; Cleveland Clinic Abu Dhabi, Abu Dhabi, Abu Dhabi, United Arab Emirates

## Abstract

**Background:**

A ventricular assist device (VAD) is either a destination therapy or a bridge to heart transplant for patients with end-stage heart failure. Unfortunately, VAD infections are a major complication postimplant. We aimed to evaluate the incidence, risk factors, and outcomes for patients with VAD infections.

Baseline characteristics and risk factors for patients implanted with ventricular assist device.

**Figure:**
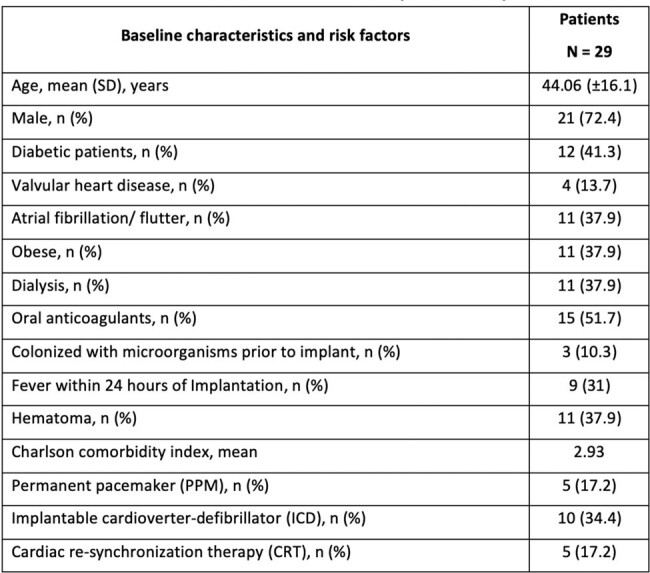

**Methods:**

We performed the single-center, observational, retrospective study on all patients who underwent VAD implantation between May 2016 and August 2022 with a minimum of 12-month follow-up post-placement. We defined VAD infections according to the International Society of Heart and Lung Transplantation (ISHLT) criteria, classifying infections into VAD-specific and VAD-related. We included all patients above 18 years of age with VAD and excluded patients who underwent VAD implantation outside our facility.

**Results:**

We included 29 VAD patients, of which 16 were HeartMate 3 left ventricular assist devices (LVADs), 12 were HeartWare ventricular assist devices (HVADs), and one was a biventricular assist device (BiVAD). We reported an overall incidence of 41.3% (n=12/29) of VAD infections during the study period. Within one year post-implantation, we had two VAD-specific driveline infections, which isolated *Pseudomonas aeruginosa* and methicillin-sensitive*Staphylococcus aureus* from the pus culture, and seven VAD-related infections (mediastinitis and bloodstream infections). Bloodstream infections included coagulase-negative staphylococci (n=4), *Klebsiella pneumonia* (n=1), and*Candida tropicalis* (n=1). We also had three patients with driveline infections one year after implantation. There were 15 patients requiring extracorporeal membrane oxygenation (ECMO) and one on Impella®. The mean (SD) length of stay during VAD infection was 56.5 (±37.6) days, all-cause mortality for patients with VAD infections was 41.6% (n=5/12), and the overall all-cause mortality in our study was 44.8% (n=13/29).

**Conclusion:**

The VAD infections for patients on additional cardiac implantable electronic devices, multiple device reinterventions, or requiring temporary mechanical support complicate the overall outcome. Further studies with a larger population from the region are necessary to make better decisions about preventing and treating VAD infections.

**Disclosures:**

**All Authors**: No reported disclosures

